# Discovery of an oviposition attractant for gravid malaria vectors of the *Anopheles gambiae* species complex

**DOI:** 10.1186/s12936-015-0636-0

**Published:** 2015-03-20

**Authors:** Jenny M Lindh, Michael N Okal, Manuela Herrera-Varela, Anna-Karin Borg-Karlson, Baldwyn Torto, Steven W Lindsay, Ulrike Fillinger

**Affiliations:** Department of Chemistry, Royal Institute of Technology, SE-100 44 Stockholm, Sweden; Disease Control Department, London School of Hygiene & Tropical Medicine, London, WC1E 7HT UK; Behavioural and Chemical Ecology Department, International Centre of Insect Physiology and Ecology, 00100 Nairobi, Kenya; School of Biological & Biomedical Sciences, Durham University, Durham, DH1 3LE UK

**Keywords:** Malaria, *Anopheles gambiae s.l*, Oviposition behaviour, Attractant, Cedrol, Attract and kill

## Abstract

**Background:**

New strategies are needed to manage malaria vector populations that resist insecticides and bite outdoors. This study describes a breakthrough in developing ‘attract and kill’ strategies targeting gravid females by identifying and evaluating an oviposition attractant for *Anopheles gambiae s.l*.

**Methods:**

Previously, the authors found that gravid *An. gambiae s.s.* females were two times more likely to lay eggs in lake water infused for six days with soil from a natural oviposition site in western Kenya compared to lake water alone or to the same but autoclaved infusion. Here, the volatile chemicals released from these substrates were analysed with a gas-chromatograph coupled to a mass-spectrometer (GC-MS). Furthermore, the behavioural responses of gravid females to one of the compounds identified were evaluated in dual choice egg-count bioassays, in dual-choice semi-field experiments with odour-baited traps and in field bioassays.

**Results:**

One of the soil infusion volatiles was readily identified as the sesquiterpene alcohol cedrol. Its widespread presence in natural aquatic habitats in the study area was confirmed by analysing the chemical headspace of 116 water samples collected from different aquatic sites in the field and was therefore selected for evaluation in oviposition bioassays. Twice as many gravid females were attracted to cedrol-treated water than to water alone in two choice cage bioassays (odds ratio (OR) 1.84; 95% confidence interval (CI) 1.16-2.91) and in experiments conducted in large-screened cages with free-flying mosquitoes (OR 1.92; 95% CI 1.63-2.27). When tested in the field, wild malaria vector females were three times more likely to be collected in the traps baited with cedrol than in the traps containing water alone (OR 3.3; 95% CI 1.4-7.9).

**Conclusion:**

Cedrol is the first compound confirmed as an oviposition attractant for gravid *An. gambiae s.l*. This finding paves the way for developing new ‘attract and kill strategies’ for malaria vector control.

**Electronic supplementary material:**

The online version of this article (doi:10.1186/s12936-015-0636-0) contains supplementary material, which is available to authorized users.

## Background

Mosquitoes of the *Anopheles gambiae* species complex (*An. gambiae sensu lato* (*s.l.*)) including *An. gambiae sensu stricto* (*s.s*.) and *Anopheles arabiensis* are among the most efficient vectors of malaria on the planet and are responsible for most deaths from this disease in sub-Saharan Africa [1]. The most effective way to prevent malaria to date is vector control. The interventions used to reduce vector numbers primarily target host-seeking mosquitoes indoors [2,3]. While these interventions are effective, increasing evidence suggests that malaria elimination is not achievable by these methods alone since residual malaria transmission is maintained by vectors that feed and rest outdoors or feed on animal hosts [4]. The development of an efficient attract-and-kill strategy for oviposition site-seeking females could be one of the novel vector control tools that is urgently called for [5,6].

To date, there has been little research investigating how *An. gambiae s.l.* females find and choose oviposition sites. It is known that water vapour helps to guide them [7,8], however, in nature many aquatic sites remain uncolonized suggesting that some are more attractive to gravid females than others [9-11]. Recently, the authors found that mosquitoes were two times more likely to lay eggs in lake water infused for six days with soil from a natural oviposition site in western Kenya compared to lake water alone in two choice egg count cage bioassays. This preference was lost when the infusion was autoclaved [12] suggesting that volatile chemicals, rather than visual cues attracted the mosquitoes. Although a number of chemicals have previously been proposed as oviposition semiochemicals for *An. gambiae s.s.* [13-15], none of these have been shown to attract gravid females over a larger distance (more than a few cm) in laboratory, semi-field or field settings.

Here, volatiles released from autoclaved and unmodified soil infusions, and the lake water used as control in the study by Herrera-Varela and others [12] were analysed. One of the compounds was selected for evaluation in: i) two choice egg-count cage bioassays to test for preferential egg-laying; ii) large semi-field systems with free-flying females to test for attraction over larger distances; and, iii) under natural field conditions. Through these experiments the first confirmed oviposition attractant for gravid *An. gambiae s.l* is described.

## Methods

### Volatile collections from soil infusions

All glassware used was first washed with an odourless detergent (Teepol, general purpose detergent, Teepol Industries, Nairobi, Kenya) rinsed in water and acetone and then placed in an oven at 200°C for at least two hours before use. Volatiles released from lake water, autoclaved and unmodified six-day old soil infusions were collected in parallel with behavioural cage bioassays previously published [12]. All the unmodified infusions elicited higher oviposition responses than the lake water or the autoclaved infusion in these bioassays [12]. Infusions were prepared by mixing 15 L of lake water with 2 kg of soil sourced from a natural *Anopheles* breeding site, located within the compound of the International Centre of Insect Physiology and Ecology-Thomas Odhiambo Campus (icipe-TOC) at Mbita, western Kenya (0°26′06.19″ South; 34°12′53.12″ East; altitude 1,149 m). The soil was collected and sun-dried for one day prior to preparation of the infusion. On the day of the experiment the infusions were sieved through clean pieces of cotton cloth to remove large debris from the soil. One half of the infusion was autoclaved at 120°C for 20 minutes and left to cool to ambient temperatures. Volatiles were collected on Tenax traps made from GERSTEL-Twister Desorption glass liners (GERSTEL, Muelheim an der Ruhr, Germany), glass wool (Supelco, Bellefonte, PA, USA) and 25 mg of Tenax**®** TA polymer (60–80 mesh, Supelco, Bellefonte, PA, USA). The traps were washed with 3 ml of methyl-tert butyl ether (MTBE, Sigma-Aldrich, Steinheim, Germany) the openings covered with polytetrafluorethylene (PTFE) tape and kept in an oven at 50°C for at least two hours before use. Dynamic headspace collections were performed from 300-ml aliquots of the three sample types in 500-ml conical borosilicate glass Erlenmeyer flasks with 24/29 sockets (Quickfit® glassware). Forty-five grams of sodium chloride (NaCl, ≥99.8%, Sigma-Aldrich, Steinheim, Germany) were dissolved in all aqueous samples before volatile collections to improve the release of volatile chemicals [16,17]. E-flasks were fitted with gas wash bottle heads and charcoal-filtered air was pumped at 100 ml/minute through the inlet and drawn out at the same speed through the Tenax trap over 20 hours after which the traps were stored at −70°C. Empty bottles sampled the same way served as control for background compounds. Volatiles were collected in parallel from empty bottles, lake water and duplicates of soil infusions (autoclaved and non-autoclaved). This was repeated over seven rounds.

### Analysis of soil infusion volatiles

The gas-chromatograph-mass spectrometer (GC-MS) system consisted of a 7890A GC (Agilent Technologies, Santa Clara, CA, USA) fitted with a 30-m long HP-5MS column (Agilent Technologies) with an inner diameter of 0.25 mm and 0.25 μm film thickness coupled to a 5975C MS (Agilent Technologies) with electronic ionization set at 70 eV, the ion source at 230°C and the quadrupole at 150°C. Tenax traps were thermally desorbed in a GERSTEL thermal desorption unit (TDU, GERSTEL, Muelheim an der Ruhr, Germany) initially held at 20°C and then increased at 120°C/minute to 250°C, the end temperature was held for five minutes. The volatile chemicals were transferred in splitless mode to a cooled injection system (CIS) injector fitted with a Tenax liner (GERSTEL). The CIS injector was held at 10°C during the TDU programme and was then heated at a rate of 12°C/second to 260°C during which the volatiles were transferred to the column in a splitless mode. Helium was used as carrier gas at a pressure of 34 psi. The temperature of the GC oven was held at 40°C for one minute and then increased by 4°C/minute to 260°C and kept there for three minutes.

Heptyl acetate (35 ng, SAFC, Sigma-Aldrich, Steinheim, Germany) in Methyl tert-butyl ether (MTBE) was injected as external standard with each sample. A hydrocarbon standard with the C8-C20 compounds (10 ng of each in cyclohexane) was run and used to calculate Kovats retention indices (RI). GC-MS data from the lake water and soil infusion samples were compared to those of the empty bottle controls for each round. All peaks that were present in the samples (both duplicates for the soil samples) and had a different retention time and/or mass spectra compared to the empty bottle control were manually integrated. Volatiles with a peak-area at least twice as big in the sample compared to the control were also included. The peak-area of the control was subtracted from the peak-area of the sample when a volatile was present in both chromatograms. The area of each integrated peak was normalized against the area of the external standard heptyl acetate injected with each sample and Kovats retention indices (RI) calculated (Additional file 1). Peaks with similar RI and mass spectra where given the same compound identification number (ID). Mass spectral data were compared using to the electronic mass spectral library, NIST 2008 for a tentative identification.

### Identification of cedrol in the soil infusion samples with authentic standard

The identity of ID 276 was confirmed using an authentic standard: (+)-cedrol, ≥99.0% sum of enantiomers, GC, optical activity α_D_^20^ + 10.5 ± 1° (Sigma-Aldrich, Steinheim, Germany). The compound was diluted in MTBE to 0.8 mg/ml and 1 μl was injected in a CIS-injector, set to a splitless mode, held at 40°C for 0.5 minutes and then heated at a rate of 12°C/second to 260°C. All other GC-MS parameters were as for the soil infusion samples above.

### Standard curve for cedrol

Eight different amounts (0.008, 0.016, 0.032, 0.08, 0.1, 0.2, 0.4, 0.8 μg) of cedrol ≥99.0% (sum of enantiomers, GC, Sigma-Aldrich, Steinheim, Germany) dissolved in MTBE were injected in preconditioned Tenax traps in the TDU unit on the GC-MS system (described above). All settings and temperature programmes were as described above for the soil infusion samples. The area of the peaks was utilized to create a standard curve, which was used to calculate the amount of cedrol collected in the soil infusion samples.

### Screening of volatile collection samples from field sites

Water samples were collected from 116 natural water bodies (puddles, pools, ponds, drains, swamps, and pits) on Rusinga Island, western Kenya (0°24′33.08″ South; 34°10′14.84″ East; altitude 1,377 m), during the long rainy season in 2012. Water samples were filtered into 250-ml wide-neck polypropylene bottles (Thermo Scientific, UK) through a clean piece of cotton cloth to remove large debris and transported in a cool box to the laboratory. The samples were transferred into 500-ml E-flasks. Volatiles in the headspace above the water samples were collected on polydimethylsiloxane/divinylbenzene (PDMS/DVB) solid-phase microextraction (SPME) fibres (65 μm Stable Flex™, Supelco, Bellefonte, PA, USA) for 20 hours. A bottle containing distilled water, stored, transported and sampled the same way as the field samples, served as control for background compounds. SPME fibres were analysed immediately after volatile collection on a GC-MS system with the same instruments, GC-column and settings as described above. The GC injector was kept at 250°C in a splitless mode; helium with a flow of 1.2 ml/minute was used as carrier gas. The oven temperature programme started at 40°C for three minutes followed by an increase of 5°C per minute to 260°C which was held for three minutes.

The GC-MS files where screened for the main ions of the four compounds closely associated with the unmodified soil infusion samples in the principal component analysis (PCA) (compound IDs 51, 263, 276 (cedrol) and 286). Only cedrol was found. The amount of cedrol in the field samples was often close to the detection limit of the volatile collection method. Hence, all samples with a peak containing two of the main mass spectra ions of cedrol (95 and the compound specific 150) at the retention time that matched cedrol were scored as positive for the compound.

### Mosquito preparation

Laboratory and semi-field experiments were carried out with insectary-reared *An. gambiae s.s.* (Mbita strain) supplied by the mosquito insectaries at icipe-TOC, Mbita, and reared following standard operating procedures. Gravid mosquitoes were prepared by selecting 300 female and 300 male mosquitoes, two to three days old, from their rearing cages at 12.00 hours and keeping them in 30x30x30-cm netting cages at 25-28°C and 68-75% relative humidity. To avoid mosquito desiccation, folded cotton towels, saturated with tap water were placed over the cages. Mosquitoes were starved of sugar for seven hours before blood feeding and allowed to feed on a human arm for 15 minutes at 19.00 hours. Afterwards unfed female mosquitoes were removed from the cages. Mosquitoes were then provided with 6% glucose solution *ad libitum.* This procedure was repeated the following day. Fed female mosquitoes were kept together with males for two days after the second blood meal and used on the third day for experiments (i.e., four to five days after first blood meal). At 16.30 on the day of an experiment visually presumed gravid females (enlarged, pale white abdomen) were selected from the holding cage [12].

### Preparation of cedrol solutions for bioassays and field experiments

Stock solutions of 10,000 ppm cedrol in ethanol were prepared by adding 150 mg of (+)-cedrol (≥99.0%, sum of enantiomers, Sigma-Aldrich) to 15 ml of absolute ethanol (puriss. pa, absolute, ≥99.8% (GC), Sigma-Aldrich). Dilutions were made by adding the appropriate amount of stock solution to lake water. For example, to make a 5-ppm solution of cedrol in water, 3.5 mL of the stock solution was added to 7 L of lake water; for each round 2.5 L of this was used for cage bioassays and 4.5 L for semi-field experiments with free-flying mosquitoes. The same formulation procedures were used to create 5-ppm cedrol preparations for all traps in the field.

### Dual-choice cage bioassays to study substrate preferences

**Figure 1:**
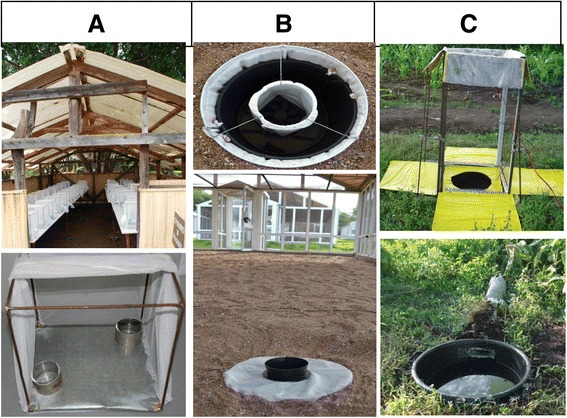
**Experimental set-up. (A)** Cage bioassays with individual gravid females under ambient conditions in makeshift huts; **(B)** Modified BG sentinel traps in a semi-field system; **(C)** Field set-up of square of electrocuting nets (up) and OviART gravid trap (down).

Experiments were done in previously described [12] make-shift sheds at icipe-TOC (Figure 1A). All experiments were carried out at ambient conditions of temperature, humidity (mean daily temperature 27 ± 5°C, relative humidity 55 ± 10%) and light. Each cage (30x30x30 cm) had two glass cups (Pyrex®, 100 ml, 70 mm diameter) covered with a metal ring and filled with 100 ml of either the control or test water. The control water was lake water pumped from Lake Victoria, stored in a settlement tank and drawn from a tap. The test water was the same water treated with the respective concentration of cedrol. The position of the test cups were randomly allocated to one of the four corners of a cage and alternated between adjacent cages to control for possible position effect. One control cup was added in each cage diagonal to the test cup to complete the two choice set-up. Five treatments were tested in parallel: 1) two untreated cups of lake water in a cage which served as the reference group; 2) lake water (control) *versus* lake water treated with 2.5 parts per million (ppm) cedrol (test); 3) control *versus* 5 ppm test; 4) control *versus* 10 ppm test; and, 5) control *versus* 20 ppm test. Cage experiments were implemented over 15 rounds with fresh cedrol stock solution and different batches of mosquitoes for every round. Fifteen to 25 replicate cages per treatment were set up per round. Cages were set at a minimum distance of 30 cm. A single gravid female was introduced per cage at 18:00. The next morning between 08.00 and 09.00 the absence or presence, and the number of eggs was recorded for the control and test cup in each cage. Non-responders (mosquitoes that did not lay eggs) were removed from the analysis.

### Semi-field experiments with free-flying gravid mosquitoes to study attraction and odour discrimination

Experiments designed to evaluate attraction (defined as the oriented movement of an insect to the source of a chemical cue from a distance of several metres [18]) of free-flying gravid female *An. gambiae s.s.* were done in a screened semi-field structure (10.8 m long × 6.7 m wide × 2.4 m high) at icipe-TOC, using modified BG-Sentinel mosquito traps (Biogents AG, Regensburg, Germany). The BG-Sentinel mosquito traps were sunk into the sand and a plastic container inserted to hold 4.5 L of aqueous solutions (Figure 1B, [19]). Two traps were set 1.5 m away from the shorter wall of the semi-field system so that they were 4.5 m apart and equidistant to the mosquito release point, 9.5 m away towards the opposite short wall. Treatments were randomly allocated to four possible corners in a randomized complete block design. Two-hundred gravid mosquitoes were released per round over 12 rounds. Mosquitoes were introduced at 18:00, about five minutes after the BG-Sentinel traps were started. Gravid mosquitoes that oriented towards either trap were sucked into a catch bag in the trap.

The peak oviposition time of the caged *An. gambiae* s.s. is between 19:00 and 21:30 [8]. To be able to compare the oviposition response within this time period to the remainder of the night the catch bags were changed at 21.30 and then collected the next day between 08:00 and 09:00. Two treatments were tested: 1) two traps with 4.5 L lake water, this served as the reference group; 2) 4.5 L lake water (control) *versus* 4.5 L lake water with 5 ppm cedrol (test).

### Estimation of release rates of cedrol from bioassay cups and BG-Sentinel mosquito traps

Cage bioassays and BG-Sentinel mosquito traps were set up in the same way as during experiments. Tenax traps prepared and cleaned as above were used to collect volatiles above the oviposition cups and gravid traps. A pump was used to draw air through the Tenax traps at a speed of 100 ml/minute. Collections were made 3 cm above the water surface of untreated lake water and lake water containing 5 or 10 ppm cedrol in the bioassay cups between 17:30 and 08:30. BG-Sentinel traps were set up in the semi-field system and collections made 5 cm above the netting covering the trap where the air current leaves the trap. The BG-Sentinel traps where baited with untreated lake water or lake water containing 5 ppm cedrol. Tenax traps were changed hourly for 12 hours. Two rounds of samples in duplicates were taken for cage tests and three for semi-field tests. Tenax traps were eluted with 200 μL of MTBE containing 20 ng of β-caryophyllene (≥98.5 sum of enantiomers. Sigma-Aldrich, Steinheim, Germany) as an internal standard. The samples were analysed using the same GC-MS instrumentation, settings and programme as described for SPME fibres above. The amount of cedrol in the samples was determined by comparing peak areas to that of the internal standard and converted to per minute release rates by dividing with the collection time.

### Field assessment of trapping efficiency of wild mosquitoes with odour-baited gravid traps

Fieldwork was implemented during the end of the long rainy season in June 2014 approximately 7 km south of icipe-TOC in Kaugege location. Collecting gravid malaria vectors has never been done routinely and gravid traps have only been developed recently [19-21]. Whilst the modified BG-Sentinel mosquito traps worked well as gravid traps in the semi-field system and were therefore an obvious choice to be taken to the field for comparison with the semi-field data, they had never been tested under field conditions prior to this work. Two other novel gravid traps, a square of electrocuting nets (E-nets) [22] and the OviART gravid traps [20], had previously been developed and preliminary field tests had shown that they performed well in the study area (S Dugassa, pers comm). Therefore, E-nets and OviART gravid traps were run in parallel to BG-Sentinel traps in the field to evaluate the effect of cedrol treatment and trap type on the collection of gravid mosquitoes. The operating procedures for these devices have been published in detail elsewhere [19,20,22].

Three study sites in close vicinity to residential houses and within 200 m of the lake shores were selected. The sites were separated by between 70 and 500 m. In each site four trap locations were chosen 10–20 m apart from each other and 5 m from the nearest house. One out of the three sites was randomly selected to receive two squares of E-nets and two OviART gravid traps whilst the other two sites received BG-Sentinel traps in all four locations. The different trap types were not set simultaneously at the same site to avoid a competition between visual and chemical cues. The OviART gravid trap and the square of E-nets provide a visual stimulus with their open water surface whilst the BG-Sentinel trap relies exclusively on chemical cues released from the trap with its convection currents. However, the trap types were rotated randomly through all three sites so that the OviART gravid trap and square of E-nets were tested in all three locations. All trapping devices provided artificial oviposition sites filled with lake water; the BG-Sentinel trap contained 4.5 L whilst the OviART gravid trap and the square of E-nets contained 8 L each. At each study site half of the traps (per type) were treated with 5 ppm of cedrol whilst the other half remained untreated. Treatment location per site was allocated randomly in such a way that each location had received the test treatment twice during the test round (eight days). Cedrol treatment was done just before the traps were switched on at 17.00. Mosquitoes were collected from the traps in the morning at 06.00. All traps were freshly set up in the afternoon in the same position for eight days, then the location of the OviART gravid traps and E-nets were relocated randomly to another study site. This was repeated twice to ensure that the alternative traps (OviART and E-nets) were in each site once (three rounds of eight days).

In order to have an estimate of the mosquito population density in the area, more established host-seeking vector collections were implemented weekly in parallel to the gravid collections from 12 households a minimum of 100 m apart from each other and within 1 to 2 km from the locations of the gravid traps. Collections were made indoors in inhabited houses with CDC light traps [23] and outdoors with cattle baited traps (CBT) [24]. The two different collection methods were chose to gain a better estimate of potential malaria vectors with varying feeding and resting behaviour. Mosquitoes were morphologically identified to genus level and *Anopheles* mosquitoes to species level [25,26]. Molecular tools were used to identify members of the sibling species of the *An. gambiae* complex and the *Anopheles funestus* complex following published procedures [27,28].

### Statistical analyses

GC-MS data were explored using PCA with supplementary variables. Only volatiles present in at least four out of the seven rounds for at least one of the sample types were included in the analysis. The data was centred and standardized by volatiles prior to analysis with Canoco 5 [29].

Dual choice cage bioassays and semi-field experiments were analysed using generalized linear models with a quasibinomial distribution fitted to account for overdispersion in R statistical software version 2.13 [30]. The proportion of responses (eggs laid or females trapped) received by the test cups in cage bioassays or test traps in the semi-field systems of the experiments with two different choices were compared with the responses received by the test cups/traps in cages/semi-field systems with two equal choices (lake water in both cups/traps). It was hypothesized that gravid females presented with identical treatments respond to both cups/traps in an approximately equal proportion (p = 0.5). The statistical analysis aimed to reveal if the test treatment of interest (e.g., increasing concentration of cedrol) received an increased or decreased proportion of responses as compared to the lake water only treatment. The experiment (two-choice, equal choice) and the round of experiment were included as fixed factors to analyse their impact on the outcome. Rounds were not significantly associated with the outcome in any of the experiments and therefore removed from the final models.

Field data were analysed using generalized estimating equations (GEE) in IBM SPSS version 20. Prior to the final analysis the data was tested for significant between-group variations in trap location and study area. Only study area varied significantly and was included in the final analysis as repeated measure with an exchangeable correlation matrix. The data fitted a negative binomial distribution. Treatment and trap type were included in the model as fixed factors. Interactions were tested but no significant associations found. All reported means and their 95% confidence intervals (CIs) were estimated as the exponentials of the parameter estimates for models with no intercept included.

### Ethics

Ethical approval for this study was obtained from the Kenya Medical Research Institute’s Ethical Review Committee (Protocol no. 363 and protocol no.422).

## Results

### Identification of putative oviposition semiochemicals

**Figure 2:**
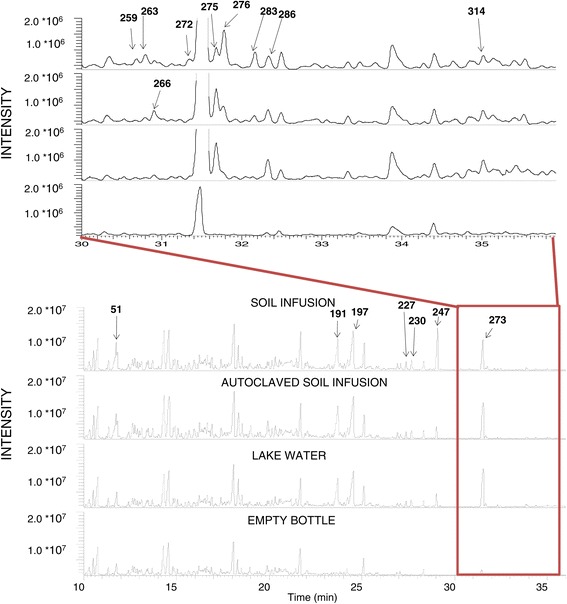
**Example chromatograms from round five of volatile collections.** One chromatogram of each sample type (unmodified soil infusion, autoclaved soil infusion and lake water) and empty bottle control. All compounds included in the multivariate analysis are marked by the corresponding ID number. Kovarts retention index (RI) and mass spectral data for each compound can be found in Additional file 1.

**Figure 3:**
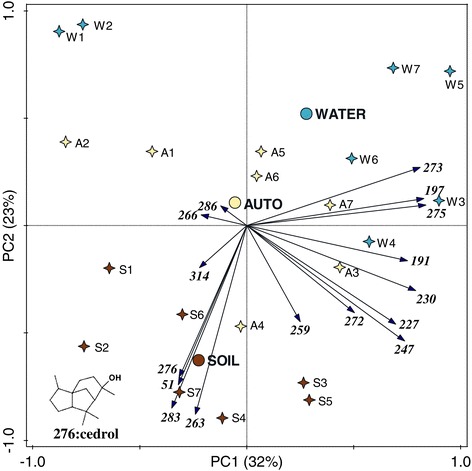
**Biplot of the GC-MS data from lake water, unmodified and autoclaved soil infusions.** The three sample types form distinct groups, mainly separated by the second principal component. Four compound IDs (51, 263, 276 and 283) group closely with the unmodified soil samples. Data from seven rounds of each sample type were centred and standardized by the volatile compounds before being subjected to principal component analysis with supplementary variables. The supplementary variables were the three sample types indicated with WATER (lake water), AUTO (autoclaved soil infusion) and SOIL (unmodified soil infusions). Each sample is indicated with a letter; W, A or S for lake water, autoclaved soil infusion and unmodified soil infusion respectively. The number following the letter indicates the round; volatiles were collected in parallel from samples with the same number.

Volatile chemicals emitted from autoclaved and unmodified soil infusions as well as the lake water were sampled in parallel to behavioural assays and analysed by GC-MS (Figure 2). Exploration of the GC-MS data using PCA indicated similarities in volatile profiles within the replicates of the same sample type but different chemical profiles between the treatments (Figure 3). Four compounds (IDs 51, 263, 276, 283) grouped closely with the unmodified soil samples. GC-MS data with volatiles emitted from water samples from natural aquatic habitats situated along the shores of Lake Victoria in western Kenya were screened for these four compounds. ID 276 was above the detection threshold in 62 of the 116 samples whereas none of the other three compounds was detected. ID 276 was identified as the sesquiterpene alcohol cedrol by comparison of mass spectral data to the NIST08 library and an authentic standard. Based on its presence in natural *Anopheles* oviposition sites and the ease of its identification, cedrol was selected for further evaluation.

Cedrol was present in all the soil infusion samples investigated (n = 14 for unmodified and autoclaved samples combined) and the amount was three times as high in the unmodified soil infusion (mean 15.8 ng, 95% CI 9.36-22.2), which was preferred for egg-laying in the previous study [12], compared to the non-preferred autoclaved infusion (mean 5.7 ng, 95% CI 4.6-6.7). In contrast, it was only detected in two out of seven lake water samples (mean of those two samples: 4.2 ng, 95% CI 3.8-4.5).

### Cedrol attracts laboratory-reared gravid *Anopheles gambiae s.s.* females

**Figure 4:**
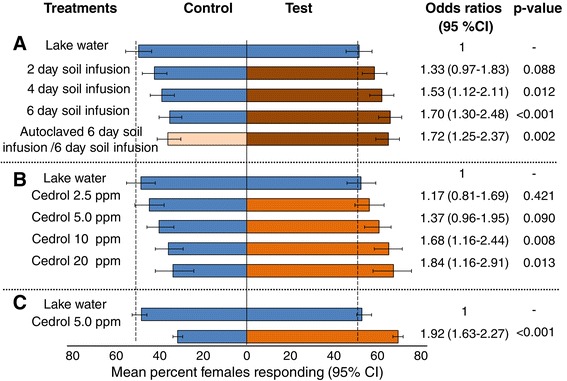
**Mean per cent of gravid** ***Anopheles gambiae*** **responding to control and test treatments in choice experiments. (A)** Cage bioassays with soil infusions of increasing incubation time and comparison of autoclaved versus unmodified infusion. The data from Herrera-Varela and others [12] have been re-analysed for this figure to show the per cent of females responding. These data present the background for the current study. Headspace collections for identification of volatile chemicals were implemented for autoclaved and unmodified six-day old soil infusions in parallel to these behavioural assays. **(B)** Cage bioassays with cedrol-treated lake water in increasing concentrations. **(C)** Semi-field evaluation of response off free-flying gravid females to cedrol-baited traps.

A series of experiments was carried out in the laboratory and semi-field with insectary-reared *An. gambiae s.s.* to determine whether gravid females respond to cedrol (Figures 1 and 4). The cage bioassays demonstrated a dose-dependent response of gravid females with increasing concentrations of cedrol increasing the probability of a female laying her eggs in the test solution. Interestingly, the dose–response matched the previously observed (Figures 4A and B) results for the soil infusions of increasing incubation time when compared to lake water.

Since these egg-count cage bioassays cannot distinguish between contact stimulants and long-range attractants [31] experiments were implemented in a large (174 cu m) semi-field system using modified BG-Sentinel traps (Figure 1B). These odour-baited traps allowed to assess the relative attractiveness of volatiles released from a trap, without the influence of visual cues or contact stimulants since the mosquitoes are prevented from seeing or accessing the test substrate. The experiments confirmed that cedrol was attractive with 69% (95% CI 66-71%) of released females collected in the treated trap (Figure 4C). The response towards the cedrol-baited trap was consistent and high from night-to-night with very little variation. Furthermore, on average 89% (95% CI 84-92%) of released gravid mosquitoes were recollected during the choice experiment when a cedrol-baited trap was present. This was in sharp contrast (p <0.001) to the experiment where both traps contained only lake water in which only 34% (95% CI 29-38%) of the released females were recollected.

The peak oviposition time of the caged *An. gambiae* used in this study has previously been determined to be between 19:00 and 21:30 [8]. In the semi-field experiment 68% (95% CI 57-78%) of the females were collected during this period, with 74% (95% CI 70-76%) choosing the cedrol-treated trap over the trap with lake water only. However, the response after 21.30 was nearly balanced, with only a slightly higher proportion of females collected in the 5 ppm test trap (58%, CI 53-62%).

Volatile headspace collections from both bioassay systems confirmed that cedrol was released from the test substrates but not from the controls. Besides the cedrol peak, no consistent difference was observed in the chromatograms from test and control treatments hence, no breakdown products of cedrol were detected. Oviposition cups treated with 5 ppm cedrol released 8.7 ng/minute (95% CI 5.9-12.7 ng/minute) and those treated with 10 ppm released 22.8 ng/minute (95% CI 18.0-29.0 ng/minute) during the 12 hours of experiment. The release rate from the BG-Sentinel traps treated with 5 ppm cedrol was on average 8.0 ng/minute (95% CI 5.4-12.0 ng/minute). Cedrol was released at consistent rates over the 12-hour experimental period with no significant difference (p = 0.293) between the peak oviposition time (19:00–21:30) and the rest of the night.

### Cedrol attracts wild malaria vectors

**Figure 5:**
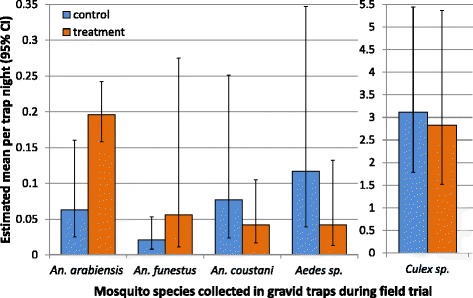
**Estimated mean number of female mosquitoes per trap night (all trap types pooled) collected during the field trial.** Error bars represent 95% confidence intervals.

Under natural field conditions a total of 933 female mosquitoes were collected in 288 gravid trap nights (12 traps per night for 24 nights); 91% were *Culex* species. Of the *An. gambiae* species complex, only *An. arabiensis* were collected in the field sites, representing 4% of the total catch. In addition, a small number (1%) of the malaria vector *An. funestus s.s.* were collected. Trap catches also included 2% of the secondary malaria vector *Anopheles coustani* and 2% *Aedes* species. Traps baited with cedrol were 3.3 times (95% CIs 1.4-7.9) more likely to trap a female *An. arabiensis* than traps containing lake water only, irrespective of the trap type (Table 1, Figure 5). However, the three trap types performed differently under field conditions with more *An. arabiensis* females caught in devices that included visual water cues like the squares of electrocuting nets and the OviART gravid trap irrespective of site and location (Table 1). Collections of host-seeking females indoors with CDC light traps and outdoors with CBTs at the same time confirmed that the overall population density of vectors in the study area was low during the study period. In CDC traps a mean of 0.73 (95% CI 0.28-1.90) and in CBTs a mean of 2.1 (95% CI 1.1-4.0) females of the *An. gambiae* complex were collected per trap night; 96% of which were *An. arabiensis*, confirming the predominance of this sibling species in the field setting.

**Table 1:** **Probability of a mosquito female being trapped in field tests**

	**Rate ratio (95% confidence interval)**				
	***Anopheles arabiensis***	***Anopheles funestus s.s.***	***Anopheles coustani***	***Aedes sp.***	***Culex sp.***
**Treatment**					
Control	1	1	1	1	1
Test	3.3 (1.4-7.9)	2.6 (0.97-6.96)	0.5 (0.3-0.8)	0.4 (0.3-0.6)	0.8 (0.7-0.9)
**Trap**					
BG	1	1	1	1	1
OviART	5.2 (0.9-30.9)	6.3 (1.6-25.4)	-^a^	-^a^	1.1 (0.5-2.3)
E-nets	10.0 (5.6-18.0)	12.4 (2.9-52.5)	12.9 (5.0-32.6)	3.5 (1.3-9.1)	8.7 (5.0-15.1)

^a^No mosquitoes trapped; factor excluded from model. Treatment: control = lake water, test = lake water with 5 ppm cedrol. Traps: E-nets = squares of electrocuting nets [22], OviART = OviART gravid traps [20], BG = modified BG-Sentinel mosquito traps [19].

Interestingly, the data indicate that *An. funestus* might show a preference for cedrol-treated oviposition sites, however due to the small sample size this result is only of borderline significance (p = 0.057, Table 1). On the contrary, *An. coustani*, *Aedes* species and the abundant *Culex* species preferred the untreated traps (Table 1).

## Discussion

This study describes the identification of the first oviposition attractant for malaria vectors of the *An. gambiae* species complex. Caged gravid females selected lake water treated with cedrol over lake water without cedrol for laying their eggs. Furthermore, the odorant attracted colonized free-flying gravid mosquitoes in large semi-field structures and increased the trap catches of wild gravid mosquitoes in the field. The attractiveness of cedrol was established in comparison to natural water from Lake Victoria which constitutes the majority of the natural, highly productive anopheline habitats in the study area [32] and which previously was found “to be the most stimulatory water treatment [for *An. gambiae*] uncovered to date” in egg-count cage bioassays [33]. This comparison is considered more realistic than one using distilled water as a comparator, since it is an artificial water source that wild mosquitoes are unlikely to encounter. It can though not be excluded that volatile compounds released from the lake water contributed to the attractiveness of cedrol. However, preliminary cage bioassays (unpublished) implemented with distilled water gave similar results as those with lake water.

The recently developed systems of analysing oviposition responses in comparison to a baseline that provides two equal, untreated choices [12], and of measuring attraction of gravid mosquitoes to oviposition substrates with modified BG-Sentinel mosquito traps [19] allowed a more detailed description of the behaviour of gravid *Anopheles* in response to odorants, since the response of individual females could be studied and stochastic effects affecting the distribution could be estimated and included in the analyses. It was shown here that cedrol not only increased the proportion of gravid females that were caught in the test trap out of the total number caught, but it also increased the proportion that responded out of the mosquitoes released. Furthermore, the presence of cedrol in the system induced a fast response, with two thirds of gravid mosquitoes trapped by 21:30.

With the ethanol-based cedrol formulation utilized here, cedrol was released in consistent rates over the entire 12 hours trapping period each night and therefore does not explain the nearly balanced response of gravid females to the traps in the semi-field experiment after 21.30. Less than one third of the collected mosquitoes were trapped after 21.30. It might be that these specimens were not fully gravid and therefore responded to high humidity to locate a resting place rather than to locate an oviposition site. For future studies, there may be value to work out better ways to formulate and dispense cedrol. The fact that it is a stable compound of relatively low volatility means that it should be well suited for development of long-lasting attractive baits.

The field study was implemented in an area of relatively low vector density as confirmed by collections of host-seeking mosquitoes. Considering that only a proportion of mosquitoes that host seek obtain sufficient amount of blood and survive long enough to become gravid, it was not unexpected that collections in gravid traps were an order of magnitude lower than catches in host-seeking traps. Despite low densities, it was three times more likely to trap *An. arabiensis* (the predominant species of the *An. gambiae* species complex in the study area) when the trap was cedrol-baited than when it only contained lake water. Previous reports from the study area show that the two sibling species *An. arabiensis* and *An. gambiae s.s.* share the same aquatic habitats [11,34,35] and therefore it is not surprising that they appear to use the same odorants for orientation and selection of oviposition sites. The collections from the gravid traps also suggested that it is worth testing the attraction of the malaria vector *An. funestus* to cedrol since a slight preference for cedrol-treated traps was recorded. Finding a semiochemical or blend that could attract gravid females of the three most important vectors of human malaria in Africa, *An. gambiae, An. arabiensis* and *An. funestus,* would represent a tremendous breakthrough for the development of novel interventions. The fact that *Anopheles* were caught in an area with very low densities and that cedrol attracted *An. arabiensis,* a vector that is becoming increasingly important in areas where indoor interventions have impacted mosquito densities, indicates a promising future for the development of an odour-baited surveillance tool [24,36].

The results presented here confirm that the modified BG-Sentinel mosquito traps work extremely well under semi-field conditions but were less effective in the field. It is hypothesized that visual cues interact with olfactory signals [37], explaining the better performance of traps with open water surfaces in the study. Further understanding of the interaction between visual and chemical cues which may result in more effective traps will increase the possibility to efficiently lure vectors into oviposition traps when competing with natural oviposition habitats.

Cedrol-treated lake water, attracted similar proportions of gravid females in the semi-field experiments as the soil infusions from which it was identified [19]. To achieve this, a release rate of cedrol, which was much higher than from the natural source, was required. A lower concentration of cedrol might be enough to attract gravid malaria vectors if released in combination with other attractants. For instance, blends of synergistic attractants have been shown to be essential for effective trapping of host-seeking *Anopheles* mosquitoes [38-40]. The analysis of the GC-MS data suggests another four putative semiochemicals, yet to be identified, that may play a role in the attractiveness of the six-day old soil infusion to gravid mosquitoes however, in contrast to cedrol none of these could be detected in the samples from natural oviposition sites in Kenya.

Cedrol is a sesquiterpene alcohol best known for its presence in the essential oil of conifers, especially in the genera *Cupressus* and *Juniperus*. However, it has been found in a large variety of plants including *Sorghum* [41], *Artemisia* [42] and swamp grasses of the genus *Cyperus* [43], which are all common in the study area. Sesquiterpenes are also known metabolites of fungi and to some extent bacteria [44-46]. It was shown here that the amount of cedrol released from a soil infusion was higher than from the same infusion that had been autoclaved and previously that the oviposition preference increased with increasing incubation time of the infusion [12]. This suggests that the release of cedrol is associated with microbial activity, possibly by metabolism of plant products. Finding the source of cedrol might further elucidate why *An. gambiae s.s.* and *An. arabiensis* prefer to lay eggs in habitats containing this compound and might help predict habitat selection and guide malaria vector control interventions.

## Conclusions

This study provides evidence that gravid females of the *An. gambiae* complex can use attractive chemical cues when orienting towards potential oviposition sites. The findings demonstrate for the first time that these chemical cues can be exploited for trapping female malaria vectors. The discovery of an oviposition attractant provides prospects for novel ecological studies and is an important breakthrough in developing ‘attract and kill’ strategies against gravid malaria vectors. This could provide a novel tool in targeting residual malaria transmission in areas where current gold-standard indoor vector control interventions are applied at full coverage but are not enough to eliminate malaria [4,6].

## Additional file

Additional file 1:
**Kovarts retention index and example mass spectra of volatile compounds included in the principle component analysis in Figure** 1 **.**
