# Supplementary material for: Discovery of an oviposition attractant for gravid malaria vectors of the Anopheles gambiae species complex
Source: Malar J. 2015 Mar 20;14:119. doi: 10.1186/s12936-015-0636-0 (PMC4404675; doi:10.1186/s12936-015-0636-0)
Supplement: Additional file 1: — Kovarts retention index and example mass spectra of volatile compounds included in the principle component analysis in Figure 1 . [file 12936_2015_636_MOESM1_ESM.docx]

| **ID** | **Retention Index** | **Mass Spectra ions (relative intensity)**  (up to 8 fragments included) |
| --- | --- | --- |
| 51 | 1034 | 83(100), 43(78), 55(54), 41(40), 56(40), 39(24), 98(19), 127(18) |
| 191 | 1362 | 71(100), 43(75), 83(37), 56(33), 41(25), 98(23), 89(23), 55(22) |
| 197 | 1386 | 71(100), 56(80), 89(73), 43(29), 41(29), 57(20), 55(19), 73(19) |
| 227 | 1473 | 177(100), 41(58), 220(48), 67(42), 135(42), 57(36), 149(32), 43(29) |
| 230 | 1481 | 55(100), 41(92), 43(87), 69(83), 83(71), 56(71), 57(68), 70(65) |
| 247 | 1524 | 191(100), 57(20), 206(16), 192(15) |
| 259 | 1576 | 43(100), 71(93), 58(69), 41(54), 57(51), 99(42), 55(40) |
| 263 | 1580 | 168(100), 143(38), 83(24), 113(22), 43(19), 59(14) |
| 266 | 1583 | 115(100), 41(35), 43(34), 57(33), 55(23), 56(19), 42(19), 69(17) |
| 272 | 1597 | 83(100), 69(94), 55(80), 41(51), 43(47), 57(41), 101(25) |
| 273 | 1603 | 71(100), 43(48), 41(12), 56(8) |
| 275 | 1608 | 57(100), 56(55), 41(45), 85(41), 43(34), 83(30), 71(30), 55 (28) |
| 276 | 1611 | 95(100), 150(72), 151(63), 43(61), 41(49), 81(40), 69(35), 107(31) |
| 283 | 1624 | 168(100), 83(54), 70(32), 113(27), 43(23), 98(22), 55(19), 41(18) |
| 286 | 1630 | 43(100), 60(82), 41(67), 102(64), 200(46), 201(42), 55(42), 183(42 |
| 314 | 1721 | 57(100), 43(66), 71(58), 41(48), 55(44), 45(40), 85(38), 69(31) |

**Additional File 1** Kovarts retention index and example mass spectra of volatile compounds included in the principle component analysis in Figure 1
